# Barriers to Contraceptive Use Among Urban Adolescents and Youth in Conakry, in 2019, Guinea

**DOI:** 10.3389/fgwh.2021.655929

**Published:** 2021-07-15

**Authors:** Nafissatou Dioubaté, Hawa Manet, Charlotte Bangoura, Sidikiba Sidibé, Mariama Kouyaté, Delphin Kolie, Alison M. El Ayadi, Alexandre Delamou

**Affiliations:** ^1^Maferinyah National Center for Training and Research in Rural Health (CNFRSR), Forécariah, Guinea; ^2^Africa Center of Excellence for Prevention and Control of Communicable Diseases (CEA-PCMT), University Gamal Abdel Nasser, Conakry, Guinea; ^3^Department of Obstetrics, Gynecology and Reproductive Sciences, University of California, San Francisco, San Francisco, CA, United States

**Keywords:** barriers, contraceptive, urban, adolescents and youth, family planning, Guinea

## Abstract

**Background:** Despite efforts to improve access to family planning, contraceptive prevalence remains relatively low among adolescents and youth in Guinea. The objective of this study was to understand the barriers to the use of modern contraceptive methods among urban adolescents and youth (15–24 years) in Conakry, Guinea.

**Methods:** This was a qualitative study using an exploratory design. It was conducted in the capital city of Guinea, Conakry in 2019. Respondents included adolescents and youth aged 15–24 years, health care providers, and parents of adolescents and youth. In-depth individual interviews (IDIs) and focus group discussions (FGDs) were used to collect the data. Sixty IDIs and ten FGDs were planned in Conakry. These data were recorded and transcribed, when applicable, from the local languages into French in an anonymous manner. The data were analyzed using a mixed (inductive and deductive) thematic approach following the elements of the socio-ecological model.

**Results:** Overall, 56 IDIs and 10 FGDs were conducted with 136 participants and included in this analysis. Respondents were adolescents (16%), youth (30%), and key informants (54%) who were health care providers (public and private), decision-makers, parents of adolescents and youth, and neighbors. Among adolescent respondents, 75% were female, and of the youth, 61% were female. Our analysis indicates various and interrelated barriers that limit the access and use of contraceptives by adolescents and youth. These included the individual (fear of side effects, cost, and rumor-related misinformation), interpersonal or family (spouse perception and sexuality taboo and perception of sexual activity before marriage), sociocultural (religious prohibitions and ethnicity), and health care system (breakdown of contraceptive methods in public health facilities, perception of service delivery, provider attitudes, visiting hours, geographic proximity of services, and quality of training received by health care providers) barriers.

**Conclusion:** In our context, the use of modern contraceptive methods by adolescents and youth is influenced by an interaction of various barriers, including individual, interpersonal, sociocultural, and health care system factors. Strengthening contraceptive uptake interventions by involving different stakeholders, including adolescents, parents, religious, and community leaders, and improving the quality of sexual and reproductive health services would help in reducing barriers to contraceptive use among adolescents and youth.

## Background

Sexual and reproductive health issues, including unwanted pregnancies, remain a significant public concern in sub-Saharan Africa (1, 2). According to a report published in 2016 on the “Costs and Benefits of Meeting the Contraceptive Needs of Adolescents,” an estimated 21 million girls between the ages of 15 and 19 become pregnant each year in developing countries, and about 12 million of them give birth to children (3, 4). At least 10 million of these young people face unwanted pregnancies each year in these countries (3). However, complications during pregnancy and childbirth are the leading cause of death for girls aged 15–19 worldwide (5, 6).

In 2019, more than 842 million women of childbearing age were using contraceptive methods, and 270 million women worldwide did not have access to contraceptive methods they needed (7, 8). However, less than half of the need for family planning (FP) was met in sub-Saharan African countries (3).

In addition, adolescents and youth often face difficulties in accessing contraceptive services (9, 10). The results of some studies conducted, particularly in developing countries, on the obstacles related to the use of modern contraceptive methods among adolescents and youth suggest that particular attention should be paid to individual difficulties, interpersonal, community, or cultural influences (11–20). Adolescent girls also face many barriers, including fear, embarrassment, lack of knowledge, and cost of services, limiting the use of these methods (11, 12, 21, 22). Furthermore, factors such as quality of and access to health services or age restrictions when seeking FP services influence adolescents and youth access and use of contraceptive methods (23–26).

It is also possible that with the demographic explosion and rapid urbanization of African capitals, young people find it more difficult to access health services in these areas because of the low adaptive capacity of health systems, including low geographic access in health areas, poor quality of services due to the lack of training and equipment, inadequate services for adolescents and youth, shortages of contraceptive methods, especially in private health facilities, etc. (27). Studies have also reported health care provider bias, and community judgment toward adolescents and youth when seeking FP services, as well as concerns about confidentiality (13, 14, 28, 29).

In Guinea, adolescence is defined as the period of age between 15 and 19 years. This period corresponds to the moment that sexuality begins (30, 31). Out of the 12 million inhabitants that the country accounted for in 2019, about 2 million live in Conakry, and of these, 16% are adolescents and youth, making it a significant subpopulation (31). In the past years, many efforts have been undertaken to improve FP in Guinea, including the creation of FP access points in health areas by integrating FP into reproductive health services (post-partum care, post-abortion care, expanded program of immunization, nutrition, etc.), the extension of the community-based service approach to rural and peri-urban areas, the organization of special FP days and integrated campaigns, the strengthening of the training program and equipment, and the reinforcement of the capacity of 25% of the providers to be able to offer FP services adapted to teenagers and young people. Despite these efforts, modern contraceptive prevalence is low in Guinea (11%). This prevalence varies among sexually active young women, ranging from 10.3% among those aged 15–19 to 11.4% among those aged 20–24 in 2018 (30). However, in the same year, 20% of 15–24-year-olds had an unmet need for family planning (27, 30).

It is therefore relevant to understand the barriers to the use of modern contraceptive methods among adolescents and youth. This information can help to significantly reduce unwanted pregnancies and their consequences, but also promote their well-being and improve their living conditions not only now but also in the future (32). The purpose of this study is to explore the perception of personal, interpersonal, community, and health system barriers that influence the use of modern contraceptive methods among sexually active urban adolescents and youth in Conakry, Guinea.

## Methods

### Study Framework

The socio-ecological model was used to deconstruct our finding on barriers of contraceptive use among adolescents and youth in urban Guinea (32). This model describes the access and utilization of modern contraceptives as a result of four inter-related factors. The first comprises individual factors such as age, gender, and economic status. The second layer includes interpersonal factors such as the nature of the relationship between two or more people, including friends, parents, communities, and health workers. The third includes sociocultural factors such as religion, customs/cultural behavior. The last layer is related to organizational and health system factors such as the availability and quality of services, including contraceptive methods.

These different factors interact in complex ways and influence access to modern contraceptives and the behavioral outcome of their use among adolescents and youth (13).

### Study Design

This was a qualitative study using an exploratory design. Data were collected for six months from June to December 2019.

### Study Site

**Figure 1:**
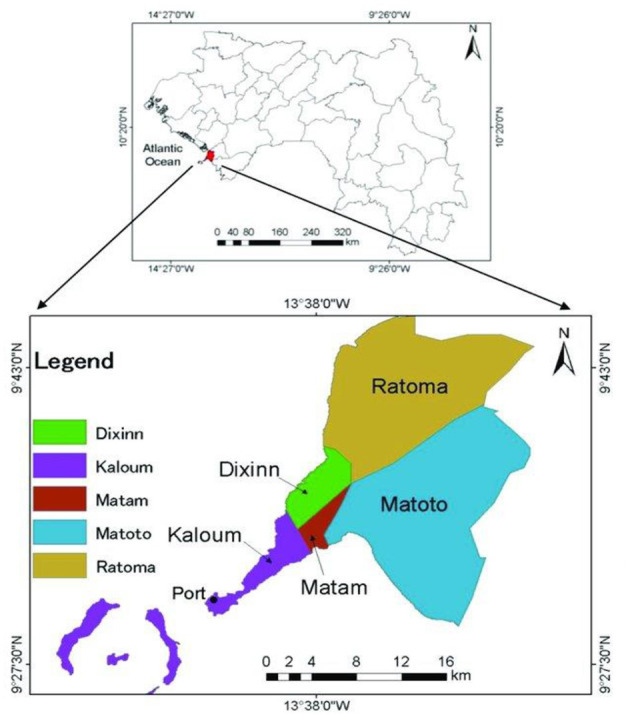
Study sites, Conakry, Guinea (33).

This study took place in the five communes of the capital city of Guinea, Conakry. Guinea is a country located in West Africa with a population estimated at 12 million inhabitants in 2012, 52% of whom are women (31). The city of Conakry has 2 million inhabitants (16%) of the national population, 26% of whom are between 15 and 24 years (15, 31). The Guinean capital is subdivided into five communes (Kaloum, Dixinn, Matam, Ratoma, and Matoto). This study was carried out in all five communes (Figure 1).

### Sampling and Recruitment of Study Participants

Study data were collected from adolescents and youth aged 15–24, and stakeholders, including national FP program managers either working in public and private institutions, parents, or relatives of adolescents and youth.

The selection of participants was done to ensure a maximum variation; for instance, adolescents and youth were selected based on their profile (marital status, level of education, etc.). Meanwhile, the selection of stakeholders was based on their involvement in sexual and reproductive health programs for adolescents and youth in Conakry. These key informants included health care providers (gynecologist, physician, midwives, nurses, etc.) from the public and private sectors and managers of FP programs.

The selection of participants with diverse profiles helped the research team capture the perspectives and experiences of participants and, therefore, strengthen the data quality. The selection process began with stakeholder mapping for key informants, while the snowball sampling technique was used to recruit adolescents, youth, and parents (fathers, mothers, guardians, and neighbors) (34). Providers and other community influencers (leaders of women and youth associations) were first identified, interviewed, and then asked to help the research team select and conduct interviews with adolescents and youth in their community. Adolescents and youth were also asked, if possible, to introduce the research team to other groups of adolescents and youth.

### Data Collection

A total of 60 in-depth interviews (IDIs) and 10 focus groups (FGDs) with eight to ten participants per FGD were planned for this study.

Thirty IDIs were planned with adolescents aged 15–19 and young people aged 20–24, six of which were conducted in each commune. The distribution took into account some socio-demographic characteristics (marital status, age group, and education level). To understand the barriers to the use of modern contraceptive methods, 30 additional IDIs were conducted with key informants, including 20 providers (public and private), four national policymakers (two of each sex), two FP program managers (public and NGO and one of each sex), and four parents (two mothers, one father, and one neighbor). 10 focus groups (*n* = 80) were conducted with adolescents and youth from different backgrounds (two FGDs per commune). The distribution of FGDs was gender-sensitive (1 FGDs per gender per commune). The selection of these different groups was necessary to capture a wide range of perspectives and experiences, to allow for the maximum variation in the information collected, and to achieve information saturation.

Four research assistants (all of them were women) trained and experienced in conducting qualitative research collected the data. They were from health research programs and continuing education institutions. They were fluent in local dialects and used the language easy for respondents to understand (in French or local languages). The study principal investigator, a medical doctor with PhD-level training in public health, supervised the work of the research assistants.

The detailed guides (IDIs and FGDs) for the semi-structured interviews were designed using the pre-requisites of knowledge from the literature and information gathered during pre-testing of these guides (15, 27, 30, 32). The guides were then pre-tested with university students and adapted. They were first designed in French and then translated into the three main local languages spoken in Conakry (Soussou, Malinké, and Pular). The questions were designed to explore perceptions of barriers to contraceptive access and use by adolescents and youth. All participants in IDIs and FGDs were interviewed in their preferred and chosen locations (homes, schools, health facilities, offices, or youth centers).

### Data Analysis

The data collected during the IDIs and FGDs were transcribed from the local languages into French, and then the transcripts were all anonymized to ensure confidentiality. We then proceeded to the thematic content analysis of the transcripts and interpretation of the results. Two researchers manually coded the interviews separately and agreed upon the subthemes through a team consultation meeting.

### Ethical Considerations

The research protocol was approved by the National Committee of Ethics for Health Research in Guinea (CNERS) and the Institutional Review Board (IRB) of the Institute of Tropical Medicine (ITM) in Antwerp, Belgium. Prior to data collection, study objectives were explained to the participants (in French or in the local language of their convenience), then a written consent was obtained from all adult participants. For those under 18 years of age, assent was obtained after written consent from parents or guardians. Fingerprinting was done for all participants (adolescents, youth, and key informants) unable to read and write while community health workers in the area served as witnesses.

## Results

### Description of the Sample

A total of 136 people took part in the study, 56 of whom participated in the IDIs and 80 in the FGDs. In the IDIs, we interacted with 26 adolescents and youth (46%) and 30 key informants (54%). Among the participants, the adolescents (9) and youth (17) were predominantly female (65.4%) and had attended school (61.5%). Among adolescent respondents, 75% were female, and of the youth, 61% were female. More than half of the adolescents and youth were married (53.8%). Key informants were composed of nurses, midwives, medical doctors, gynecologists, parents (father, mother, or relative), and decision-makers (national, regional, and communal health directorate levels). In addition to the IDIs, 80 adolescents and youth participated in 10 FGDs, including two FGDs held in each commune of Conakry, taking into account gender (five males and five females), education level, and marital status of participants (Tables 1, 2).

**Table 1:** Characteristics of participants in individual in-depth interviews (IDIs) in the five communes of Conakry urban area.

**Socio-demographic characteristics**	**Number (*n* = 26)**	**Percentage (%)**
**Types of participants**		
Adolescents	9	16.1
Youth	17	30.4
**Key informants (*****n*** **=** **30)**		
*Health care providers*	22	39.3
*Decision-makers*	4	7.1
*Parents (fathers/mothers/guardians)*	4	7.1

**Table 2:** Characteristics of adolescents and youth participants in individual in-depth interviews (IDIs) in the five communes of Conakry urban area.

**Socio-demographic characteristics**	**Number (*n* = 26)**	**Percentage (%)**
**Ages groups: adolescents and youth**		
15–19	9	34.6
20–24	17	65.4
**Age group of male vs. female**		
Male (n = 8)		
15–19	2	25
20–24	6	75
Female (18)		
15–19	7	38.9
20–24	11	61.1
**Sex**		
Men	8	30.8
Women	18	69.2
**Residence: adolescents and youth**		
Kaloum	6	23.1
Dixinn	6	23.1
Matam	4	15.4
Ratoma	5	19.2
Matoto	5	19.2
**Schooling: adolescents and youth**		
Attended formal school	16	61.5
Did not attend formal school	10	38.5
**Marital status:- adolescents and youth**		
Married	14	53.8
Unmarried	12	46.2

Following the theoretical framework, the study results were organized according to the categories of individual, interpersonal, community, and health system barriers. Perceptions of the different types of barriers to contraceptive use among adolescents and youth were presented, taking into account the variation in these perceptions among types of participants, gender, age group, marital status, type of health facility (public or private), and residence (urban commune).

### Individual or Personal Barriers

Factors on individual barriers to contraceptive use vary from age group, marital status, and gender of respondents (adolescents and youth). These findings were contextualized by adding perceptions of key informants. Thus, three subthemes related to personal barriers to contraceptive use emerged from the interviews: costs of accessing contraceptive methods, fear of contraceptive side effects, and lack of information.

### Costs Related to the Use of Contraceptive Methods and Their Social Consequences

The cost of contraceptive methods was the first challenge mentioned by participants—both adolescents and youth and key informants. They cited the lack of financial independence of adolescents and youth as a barrier to accessing contraceptive methods. At this age, urban adolescents and youth depend on their elders. Married participants mentioned the same challenges, and the reasons given were their husbands' poor perception of the methods and of the users and their refusal to provide financial support.

In addition, consultation fees or incentives for health care providers were also mentioned by participants as a barrier to contraceptive use among adolescents/youth. In addition, geographical barriers to accessing health facilities were also mentioned, given the cost of transportation to a health facility that is located far from home.

≪*. The financial difficulties of adolescents and youth constitute a main barrier to their access to contraceptive methods since these methods are not free of charge. From condoms. implants cost more than 100,000 GNF ($10), the IUD is more expensive. you see they can't afford it [buy]*. ≫. (IDIs, Man Doctor, Public Health Structure, Commune of Kaloum).

For some participants, this financial inaccessibility to contraceptive methods is fueled by the reluctance of parents of adolescents and youth to support them financially. This parental reluctance was reportedly due to their perception that putting adolescents and youth on contraception would encourage them to engage in active and “uncontrolled” sexuality and expose them to sexually transmitted infections, including HIV/AIDS.

≪*As a father, I would never give money to one of my sons to pay for a rubber [condom]. By doing this, you encourage him to do stupid things (got a girl pregnant) outside and then bring you problems. So I look at all this, maybe when they start working*. ≫. (IDIs, Father of Adolescents and Youths, Commune of Matoto*)*.

Some adolescents and youth reported that this financial inaccessibility often pushed them to give up using their preferred contraceptive method and to switch to traditional methods such as the calendar method, abstinence, or the “necklace method” (cycle beads).

### Fear of Side Effects and Perceived Health Risks of Using Contraceptive Methods

According to adolescents and youth, and health care providers, fear of the side effects of FP was an important barrier to the use of these methods. For adolescents and youth, the onset or management of these effects, which were sometimes unknown to some, could often be perceived by their family members and thus exposed them to (negative) judgments from them. The mentioned side effects included general fatigue, nausea, insomnia, or menstrual disorders that could according to participants, last up to 10 days. For others, the consequences of these effects were infertility and therefore directly related to the possibility of having children in the future, and difficulties related to the pregnancy of the users, in particular complications during childbirth (dystocic delivery, abdominal pain, or episiotomy). This is illustrated below by an educated teenager:

≪*Because sooner or later these women will be married and they are used to taking the pills or anti ball (implant), it could become chronic and therefore they will not have children. So there are effects*≫. (FIDs, P06 Educated Adolescent, Commune of Dixinn).

However, the perception of the health risks associated with contraceptive use differs between the two age and gender groups. For some participants (adolescents), the main personal barrier to contraceptive use was a negative perception of modern contraceptive methods. Both girls and boys said that modern methods have a negative impact on the health of users (temporary or permanent infertility). This point was particularly emphasized by an unmarried teenager who stated the following:

≪*Sometimes I talk to people who say that contraceptive methods are not good for women. You see, they say that they can get sick because of these methods, for others too, if you take it, a few days or a few months later, the method can disappear, if it disappears from your arm, you can have sterility problems*≫. (IDIs, unmarried Adolescent man, Commune of Kaloum).

As for the participants (young single and married men), the use of one of the modern contraceptive methods may push adolescents to have multiple sexual partners. This may lead them to opt for a life that they described as easy, a life of debauchery (frivolity), and it was this aspect that they (single and married young men) disapproved of the use of contraceptive methods. This view is illustrated in the following verbatim:

≪*I would say that we have to organize educational focus groups to be able to give advice to our sisters, their ambition pushes them to sell themselves. it is there that they get the idea of going out with a lot of men, as they take medication there, the fear of falling (pregnant) no longer exists, so we have to advise them to keep an eye on our sisters*≫. (FGDs, Young Literate Men, Commune of Kaloum).

However, from the point of view of other respondents (young unmarried women), although the use of modern contraceptives is relevant because it has allowed them to avoid unwanted pregnancies and has encouraged them to have control over their sexual life, they needed more information on it.

### Lack of Information and Their Consequences (Rumors)

The lack of knowledge and information among adolescents and youth was also reported by participants as a factor that encourages the spread of rumors that negatively influence contraceptive use. Considering the educational level of community members, their main source of information was word-of-mouth. This source was the basis for countless (negative) rumors circulating in the study site. The rumors cited included: “Implants can disappear from the body of their users and not be found,” “Implants can cause difficulty in childbearing,” “Contraceptive methods are designed to make young African women sterile.” These two quotes from two types of stakeholders highlight this statement:

≪*We learn this (modern contraceptive method) often in the neighborhoods when we talk among ourselves wives and also with our husbands in the bedroom that when you take the medication you are not going to have children and that it can even cause illness in some people. If your daughter gets married and she can't get pregnant, her home will not be stable at all and right away it will be said that she had a life of debauchery. I will never let my daughters take this filth (with a scornful pout)* ≫. (IDIs, Mother of adolescents and youth, Commune of Matoto).

≪*In my opinion, implants are not good, because it is one thing when you take you don't know if you are going to have children or not. You see no! So why wear something that will prevent you from having children in the long run. So if you wear it and then you don't have children, it's not someone else's fault, it's your own fault* ≫. (IDIs, Literate adolescent girl, Commune of Kaloum).

### Interpersonal and Family Barriers

Three subthemes emerged from interviews with participants on interpersonal barriers to contraceptive use: perceptions of spouses of contraceptive use; the taboo nature of sexuality; and perception of any sexual activity before marriage. The results were presented by addressing different perceptions of the types of respondents included in this study.

### Perceptions of Spouses of the Use of Contraceptive Methods

Negative perceptions about the use of contraceptive methods are reflected in the sometimes outright refusal of some men to support their partners (financially or psychologically) in using a contraceptive method, often resulting in arguments within the couple. However, the participants explained that the favorable opinion and support of their partners gave them peace of mind and ease in the couple since it is generally the men who have the power to decide. Thus, many adolescents and youth (married, single, or in a relationship) stated that it is vital for them to have the approval of their partners and support to avoid possible conflict.

≪*Yes, for example, there are partners who discourage their partners from using planning methods. My husband doesn't want to hear about it (referring to planning), so I hide to take my injections every three months. If he ever finds out about this, I will have big problems in my couple*≫. (IDIs, young married woman, Commune of Dixinn)

According to this provider, it is the men (husbands) themselves who constitute the barrier to the use of contraception by women, especially among those who wear Hijabs to cover their face for religious reason.

≪ *It is the men who constitute a barrier to the use of FP among Wahhabis*≫. (IDIs, Male Doctor, Private Clinic in Koloma, Commune of Ratoma).

### The Taboo Nature of Sexuality

According to the participants, any topic related to sexuality should be avoided in parent–child discussions, including contraceptive methods. According to adolescents and youth, they refrain from discussing such topics with parents (fathers, mothers, sisters) and sometimes with their spouses because they feel that this avoids any suspicion of any sexual activity or life. However, the school (for educated respondents) and discussions between peers (friends) helped fill this gap.

≪*There is the religious side of things, and it is not part of our culture to teach children about sex and how to behave, except for those who are lucky enough to go to school, if not, they are unlikely to have information about planning*≫. (IDIs, young married woman, Commune of Matam)

In the opinion of some parents, discussions about sexuality and contraception are inappropriate with children. According to them, their children should be spared by such discussions to avoid their early exposure to sexuality.

≪*We don't want the child (youth or teenager) to be confronted with all this before they are old enough to start a family. We have to be careful with them about these things, so it's something (sexuality and contraception) that we have to hide from them*≫. (IDIs, mother of adolescent girls, Matoto commune).

### Perception of Any Sexual Activity Before Marriage

According to the participants (adolescents and youth), there was a consensus that the preservation of the virginity of girls until marriage is an honor for the family and the community. Consequently, it is inappropriate and “indecent” to engage in conversations about contraceptive methods, they said.

≪*. In our culture, girls must keep their virginity until marriage. it's a source of pride for the whole family and proof to the community that you come from a good family and are well educated*. ≫ (FGDs, young married women, commune of Ratoma).

However, parents, especially mothers, put forward another point of view concerning the beginning of sexual activity among unmarried adolescents and young. According to them, “the new generation” is in a hurry to initiate sexual activities, so advising the use of condoms (for young men) or implants (for women) to avoid pregnancies is necessary although it may have adverse implications (like reported above).

≪*You know the kids there are all in a hurry now (pointing to young people outside a tea shop). They all use plastics (referring to condoms). I have five girls at home., to avoid disappointment I don't ask them this kind of question. All I ask them is not to bring pregnancies or even the disease (HIV-AIDS) to my house. This new generation there are all taking “anti-bale” (Implant)* ≫. (IDIs, Mother of adolescents and youth girl, commune of Matoto).

### Social and Community Barriers

Different perceptions were presented in this section, and two subthemes emerged from the interviews about social and community barriers to contraceptive use. These were described in the following.

### The Prohibitions of Religion

Religious norms that “having a child is God's gift” or “sexual relations among unmarried adolescents and youth are forbidden” were perceived by participants as barriers to contraceptive use. On the other hand, some participants reported that using contraceptive methods to avoid pregnancy, even for newlyweds, is an act of “homicide” because “having a child is a gift from God” but for that, you have to wait until you are married.

≪ *The Muslim religion does not accept it; it is purely clear in the religion that trying to reduce the number of children (through the use of contraceptive methods) “equals killing the children. The use of condoms, injections, or other contraceptive methods is not permitted because the religion prohibits premarital sex. Anyone who fails to do so, will be responsible for his or her actions in the afterlife., no, among Muslims planning is forbidden*≫. (FGDs, Educated Adolescents Men, commune of Dixinn)

However, other participants reported that using family planning methods is not a religious prohibition (Muslim religion) but rather a social prohibition (imposition).

≪*It is on the religious side, they must be told that religion does not prohibit the use of planning methods among the bride and groom. And also on the social side, it is necessary to review*≫. (IDIs, Man Doctor, Public Heath Structure, Commune of Kaloum).

Also from a religious point of view, the virginity of an unmarried adolescent or young woman was considered sacred, an act of purity and a symbol of good education for her parents; it represents a source of happiness and pride that automatically confers them respect and great consideration in their community. Thus, the use of modern methods of contraception would be contradictory to virginity.

≪*Yes speaking of the Muslim religion, it is not recommended because it is strictly forbidden for a teenager to go through all these means. As long as you are not married, even as a bride, it is not recommended*≫. (IDIs, Unemployed Young Woman, Commune of Matoto).

### Cultural (Ethnic) Barrier to Contraceptive Use

According to some adolescents and youth, the use of contraception and the users are not well seen in the majority of ethnic groups (e.g., the Malinke or Peulh), especially if they are adolescents or unmarried youth. The reasons given for this were that all practices related to sexuality are taboo and that unmarried people are supposed to be pious and chaste and must therefore keep their virginity until marriage. The same opinion was shared among the key informant group, especially the health providers, but the latter emphasized the case of female sex, and therefore, those who could not wait for marriage would hide to use a contraceptive method. The two quotes below shed light on this passage.

≪*I am Malinke, not even among the Malinke only, but among the Peulhs it is also forbidden because they say to themselves that the girl must go virgin at home and being at home the decision rests with the husband*≫. (FGDs, Literates Adolescents Girls, Commune of Dixinn).

≪ *One of the difficulties in this matter (referring to the use of contraception) is the rejection of society, especially in the Peulh ethnic group where virginity is considered sacred. So for those who can't wait (until marriage), they hide and come to us for planning*≫. (IDIs, Male doctor, Hamdallaye Private Clinic, Commune of Ratoma).

### Barriers Related to the Health Care System

Views on barriers to contraceptive use related to health systems vary according to the different profiles (health providers, adolescents, and youth), experiences, and gender of the respondents in this study. They include the breakdown of contraceptive methods in public health facilities, perception of FP service provision in public health facilities, provider attitudes, the barrier of consultation time, geographic proximity to the facility, and the influence of the quality of training received by health providers.

### The Breakdown of Contraceptive Methods in Public Health Facilities

A delay in the supply chain of contraceptive methods was mentioned, which often leads to a shortage of contraceptives in public facilities offering FP services. From the users' point of view, the consequences of this unavailability were linked to the capacity of these health facilities to meet the contraceptive needs of users. However, this inability is even more important for the providers than if it were an adolescent or young person who often hid to obtain this service. The repercussion of this unavailability was the possibility for this group to abandon contraception. The following citation supports this statement.

≪*As soon as there is a breakup it's not good, you lose the person concerned. He comes once, he doesn't have the condoms, once, twice, three times. So he won't come anymore*≫. (IDIs, Woman Doctor, Gbessia Port 1 Heath Center, Commune of Matoto).

### Perception of Service Delivery in Public Health Facilities

Some adolescents and youth have a negative perception of the quality of FP services offered in public health facilities and, therefore, stated that they prefer using private health facilities. The reasons given were that they are well received in private facilities as opposed to public ones; they are less likely to meet a relative in private health facilities; they find an intimacy that allows them to express their concerns without fear; they are given time and interest, and most importantly; they are not judged in private facilities

However, some participants said that they used public FP services, but always on the recommendation of a friend who had already been satisfied. In this case, appointments were usually made in the evening to avoid meeting a relative in the health facility. Here is a quote from one provider who said:

≪ *They, they, when they have money, they wait until the evening in the hospital, there is less attendance. When they go to some doctors, they can answer outright. In the evening now, you will find that the hospital is quieter even if there are patients, everyone will be in his room. So, you can come and do what you have to do in the ward and leave*≫. (IDIs, educated young woman _Commune of Dixinn)

Providers in private facilities mentioned that they wanted to actively participate in improving FP services for adolescents and youth and that the authorities should take their cases into account. Here is a quote from a provider at a private facility that supports this:

≪ *Well, I would say that we are much more marginalized in the context of FP while youth and adolescents attend the outreach facilities better than the public facilities*≫. (IDIs, Gynecologist, CSA in Kamelya, Commune of Dixinn).

### Attitudes and Perceptions of the Health Care Provider

Among the difficulties related to health providers, some adolescents and youth first mention the lack of training of providers on FP. In their view, the religious or even personal beliefs of a trained provider, especially a woman, should not interfere with the health service she is supposed to provide. In general, their negative perceptions of users and contraceptive methods were barriers to satisfaction and often led to reprimands and criticism. Adolescents and young people said that negative perceptions of users are behind some of the rumors about FP.

≪*If it was possible to tell the doctors at the hospital not to corner people about this, that is to say not to ask why you want to do it, if you are married or not, if you have the opinion of your parents and sometimes even when we go to the hospital, there are some doctors who look at us badly*≫. (IDIs, Illiterate adolescent girl, Commune of Ratoma).

According to the health providers, the attitude of some of their colleagues, especially women, constituted a barrier to the use of contraceptive methods for these “young users,” especially if they were unmarried. The reasons attributed to this were the non-adherence to the concept of the use of contraceptive methods by unmarried people, which is sometimes reinforced by the latter's religious conviction or belief. The illustration of this provider supports this statement.

≪ *You know, a lot of our colleagues especially those who work in the public do not buy into the idea of these young people using contraceptive methods (referring to adolescents and youth). When a client comes to them (female provider) asking to be planned, if it's young people; they often tell them to study and leave FP and focus on their studies*≫. (IDIs, Nurse, CSA Berney Foteba of Tannerie, Commune of Matoto).

Also, the negative perception of the providers was often influenced by age and marital status. Another difference was perceived in the way the rich and poor were treated. Some male providers, due to a lack of training and “common sense,” used the challenges they faced (lack of money) to satisfy their personal needs, such as getting into a relationship with a female user in exchange for the free method.

≪ *For example, I met a girl, there is a doctor who wanted her. He told her that if she is afraid, he can put a contraceptive method on her arm or give her an injection, so that she doesn't get pregnant. He said that if she agreed, he would give her the medication she would choose for free* ≫. (IDIs, Single Male Teenager, Commune of Kaloum).

### Barrier of Consultation Time

Adolescents and youth mentioned their preference to access health facilities in the evenings rather than during the day. The reasons they cited were that in the evening they could only find the health care team, and the facility was less frequented by clients. This reduces the risk of meeting a relative or acquaintance on the premises of the facility.

≪*When they have the money, they wait until the evening, at the hospital, there is less attendance. …. In the evening now, you will find that the hospital is quieter even if there are sick people, everyone will be in their own room. So you can come and do what you have to do in the ward and leave* ≫. (IDIs, Unmarried Young Woman, Commune of Ratoma).

### Geographical Proximity of the Structure

Another factor influencing contraceptive use, according to some participants, was the proximity of a health care facility to their homes. They said they preferred to go to a facility in a different neighborhood than their own because they were afraid to meet a family member, acquaintance, or even a doctor that their family frequented.

≪*Some don't want to do things in their neighborhood, so they can go to a friend's house in another neighborhood to plan ahead*≫*. (*IDIs, Illiterate female Teenager, Commune of Matoto).

### Influence of the Quality of Training Received by Health Providers

According to some providers (nurses and midwives), training in adolescent and youth sexual and reproductive health, including FP, was lacking in their academic training. The reasons provided included the high number of health schools in Conakry (nurses and midwives) resulting in low recruitment and teaching standards with some health topics not taught to students. This, to them, explained the inadequate practices (bad reception, even in the services offered including FP) observed in some public health facilities.

≪*I don't even remember receiving any training on adolescent or youth sexual health since I finished health school and even then (shaking his head negatively)!* ≫ (IDIs, midwife, CSA Berney Foteba of Tannerie, Commune of Matoto)

≪*The information we have is through our readings in documents*≫. (IDIs, Doctor, Center Medical Communal Bernard KOUCHNER, Commune of Kaloum)

In addition to the lack of training, other providers reported the influence of personal values that interfered with the quality of FP services offered to married users, which were described as better than those provided to single users.

## Discussion

This study provides interesting insights into understanding the individual, family, community, and health system barriers that negatively influence the decision of adolescents to use modern contraceptive methods. Our results suggest that despite persistent negative perceptions of modern contraceptive methods of participants, individual and community norms, and health system barriers, adolescents continue their quest for knowledge and use of modern methods over traditional methods that have not been proven effective in preventing unwanted pregnancies.

Among individual barriers, study participants cited fear of side effects and consequences (infertility), cost, and misinformation related to rumors as major obstacles to using modern contraceptive methods. Fear of side effects of FP products (including health risks and consequences) was perceived as a limiting factor in the decision of girls to engage in modern contraceptive use. Also, parents or other relatives or people sometimes view this group of FP users as individuals living a “promiscuous life.” Other studies have reported that adolescents often face many barriers (fear, embarrassment, cost, and lack of knowledge) that limit the use of modern contraceptive methods (6, 11, 12, 21). In our study, side effects were closely related to the ability of users to be fertile in the future. Thus, individual and mass communication strategies that include information about the types of contraceptives, their mode of action, product-related side effects, and how to manage them may help reduce such individual barriers (15). Moreover, adolescents and youth access to contraceptive methods is a complex phenomenon, which requires the alleviation of the financial accessibility barriers in health care settings (e.g., free access to methods). In addition, some of the mothers we met argued that modern contraceptive methods are made for birth spacing among married women. Thus, parents, especially mothers of adolescents and youth, have a negative influence on their use of modern contraceptive methods. In our context, the discussion of sexuality, including the use of contraceptive methods by young (especially unmarried) girls, remains taboo. According to some parents, especially mothers, discussing sexuality or contraceptive use with their unmarried daughters may encourage them to initiate premarital sex. For some parents, for example, their culture recommends that girls keep their virginity until marriage. This may be because, in our context, where the foundations of social life are essentially built on religious and cultural norms, it is a great pride and honor for parents when their daughters keep their virginity until marriage. To ensure that parents, especially fathers, use financial dependence on this group as a means of enforcing chastity and a way to avoid unwanted pregnancies, which they describe as “nonsense and a source of financial problems and dishonor” for the family.

Interpersonal and family barriers, dominated mainly by the negative influences of women's spouses, are also a source of barriers to contraceptive use by young married women. The power of the spouse in married women's decision to use modern planning methods has been documented in other studies in Guinea (30) and elsewhere in the subregion (6, 35–37). In the plan to reposition FP in Guinea, the strategies put in place did not consider the influence of this decision-making power (27). Therefore, we recommend that men be included in strategies to improve the use of modern contraception and that both sexes (men and women) have a say in FP policies.

In addition to individual and family barriers, social and community barriers have an equally important impact on the ability of adolescents and young people to decide to use modern planning methods. We found that adherence to certain religious and community norms was a barrier to autonomy of women, particularly for adolescents and youth. These norms designate people who choose to limit births as acting against religious prescriptions. Also, they describe FP users as perpetrators of infidelity or homicide. This reflects a lack of communication between parents and their children on sexuality and reproductive health topics. For example, some parents, particularly mothers, feel very uncomfortable discussing sexuality and contraception with their own children who are of childbearing age due to cultural norms. Similar findings have been documented by Samandari et al. in Niger in 2019, Ezenwaka et al. in Nigeria in 2020, and Sanchez et al. in Nigeria also in 2021, who found barriers such as religious beliefs and lack of autonomy in contraceptive use by adolescents and youth (28, 37, 38).

## Study Limitations

One of the main limitations of our study is its qualitative nature, so it cannot be generalized to the entire population of adolescents and youth in Guinea. Thus, the various barriers identified are specific to the urban area of Conakry. In addition, because of the sensitivity of the topic on sexual and reproductive health in our context, there may be a response bias among some participants. Future studies, particularly ethnographic studies, should also be undertaken in other regions, including rural areas, to examine better the community and individual factors that negatively influence the adoption and use of modern contraceptive methods by adolescents and youth.

## Conclusion

In our context, the results of this study demonstrate that there are many barriers that adolescents and youth face in seeking a contraceptive method. To reduce the impact of these perceptions, which are still alive in popular beliefs and perpetuated in communities, priority action areas should include training of health care providers, involvement of spouses (husbands), and religious or community leaders in sexual and reproductive health and right programs. Also, adapting FP services to meet urban adolescents and youth needs must be a priority in promoting contraceptive use.

## Data Availability Statement

The original contributions presented in the study are included in the article/supplementary material, further inquiries can be directed to the corresponding author/s.

## Author Contributions

ND analyzed the data and drafted the paper which was approved and commented by all authors. All authors contributed to the article and approved the submitted version.

## Conflict of Interest

The authors declare that the research was conducted in the absence of any commercial or financial relationships that could be construed as a potential conflict of interest.
